# Preformulation and Long-Term Stability Studies of an Optimized Palatable Praziquantel Ethanol-Free Solution for Pediatric Delivery

**DOI:** 10.3390/pharmaceutics15082050

**Published:** 2023-07-30

**Authors:** Giselle Bedogni, Paula Garcia, Katia Seremeta, Nora Okulik, Claudio Salomon

**Affiliations:** 1 Instituto de Química Rosario, Consejo Nacional de Investigaciones Científicas y Técnicas (IQUIR-CONICET), Suipacha 531, Rosario 2000, Argentina; bedogni@iquir-conicet.gov.ar; 2 Planta Piloto de Producción de Medicamentos, Facultad de Ciencias Bioquímicas y Farmacéuticas, Universidad Nacional de Rosario, Suipacha 570, Rosario 2000, Argentina; paulagarcia.far@gmail.com; 3 Instituto de Investigaciones en Procesos Tecnológicos Avanzados, Consejo Nacional de Investigaciones Científicas y Técnicas, Universidad Nacional del Chaco Austral (INIPTA-CONICET-UNCAUS), Cte. Fernández 755, Presidencia Roque Sáenz Peña 3700, Argentina; kseremeta@uncaus.edu.ar (K.S.); nora@uncaus.edu.ar (N.O.); 4 Área Técnica Farmacéutica, Departamento de Farmacia, Facultad de Ciencias Bioquímicas y Farmacéuticas, Suipacha 531, Universidad Nacional de Rosario, Rosario 2000, Argentina

**Keywords:** parasitic disease, praziquantel, co-solvency, solubility, palatability, pediatric patients

## Abstract

To date, the treatment for cysticercosis and neurocysticercosis consists of a single oral intake of praziquantel (5–10 mg/kg), which since it is only available as tablets, hinders its administration to pediatric patients. Praziquantel is a poorly water-soluble drug which represents a challenge for its formulation in solution, particularly for the pediatric population. Thus, this study aimed to develop a palatable solution for praziquantel using pharmaceutical-accepted co-solvent systems. A design of experiments approach was applied to identify the optimal conditions for achieving a suitable amount of praziquantel in solution using co-solvent mixtures. Thus, praziquantel solubility increased from 0.38 up to 43.50 mg/mL in the optimized system. A taste masking assay in healthy human volunteers confirmed a successful reduction of drug bitterness after the addition of selected flavors and a sweetener. Stability studies were also conducted at different temperatures (4, 25, and 40 °C) for 12 months Even though the presence of the three known impurities of praziquantel was observed, their amounts never exceeded the acceptance criteria of the USP. Thus, this novel approach should be considered a valuable alternative for further preclinical studies considering the high prevalence of this infection worldwide.

## 1. Introduction

Human taeniasis/cysticercosis is a neglected parasitic infection caused by different species of *Taenia*. Diverse factors such as inappropriate hygiene, culinary practices, and consumption of contaminated raw or undercooked beef or meat of natural intermediate hosts facilitate the acquisition of the mentioned infection [1,2,3,4]. Even though it is frequently present in low- and middle-income countries of America, Asia, and Africa, they are re-emerging in North America and Europe due to migration movements and new habits related to pork consumption [5]. The infection caused by the larval stages of *Taenia solium* (*T. solium*) may lead to gastrointestinal pain, vomiting, diarrhea, and neurocysticercosis, which produces epileptic seizures and other neurological diseases [6,7]. In general, adults and children can be successfully treated with a single dose (5–10 mg/kg) of praziquantel (PZQ), a drug included in the List of Essential Medicines for Children [8]. However, to date, the lack of a suitable pediatric formulation hinders the treatment of the pediatric population since only PZQ tablets (150, 200, or 600 mg) are commercially available and are typically divided and crushed for dosing purposes [9]. Although splitting tablets is common practice, it may lead to low dose accuracy, low drug bioavailability, and the appearance of adverse events [10,11]. Another risk associated with splitting tablets is the unpleasant taste of the drug [12,13]. In addition, PZQ exhibits a poor aqueous solubility (0.381 mg/mL) and, according to the Biopharmaceutics Classification System, is a Class II drug (low aqueous solubility and high permeability) [14]. Therefore, its bioavailability can be erratic or incomplete, and higher doses are needed to reach therapeutic plasma concentrations after oral administration [15,16]. Recently, many efforts have been reported to solve such drawbacks. A consortium created in 2012 by Merck KGaA, Astellas Pharma Inc., Swiss Tropical and Public Health Institute and Lygature explored different strategies to develop a small, orally dispersible tablet with an acceptable taste of the biologically active L-PZQ enantiomer for the treatment of preschool-aged children with schistosomiasis [17]. Gonzalez et al. described the formulation of nanoparticulate systems of PZQ by high-pressure homogenization and further microencapsulation by spray drying techniques. Such microparticles were, then, easily redispersed into a palatable vehicle [18]. Münster et al. formulated solid lipid extrudates to encapsulate and mask the bitter taste of PZQ, using mixtures of Eudragit^®^ E PO and glyceryl monostearate at different ratios [19]. Later, Albertini et al. microencapsulated PZQ through mechanochemical activation and spray congealing methodologies to prepare a pediatric PZQ dosage form [20]. Recently, 3D-printed tablets of amorphous PZQ for pediatric patients were prepared from pellets or powder forms obtained by the hot melt extrusion process [21]. Despite all these recent and important pharmaceutical approaches, a PZQ solution for children is still lacking [22]. Liquid oral dosage forms have been recommended by different regulatory agencies taking into consideration better flexibility in terms of dosage and easier swallowing [23]. For this purpose, polyethylene glycol 400 (PEG 400), propylene glycol (PPG), and ethyl alcohol (EA) are commonly used to enhance the solubility of hydrophobic drugs [24,25]. However, finding a suitable mixture of solvents to solubilize a maximum amount of a drug may lead to an expensive and time-consuming process [26]. To speed up the process of finding the right co-solvent system, it is very convenient to use a design of experiments (DoE) approach [27]. DoE allows the evaluation and optimization of various formulation and process parameters simultaneously, determine the main effects and interactions among variables, and identify the optimal conditions for achieving desired product attributes [28]. Therefore, one of the aims of this work was to apply a DoE approach to determine an optimized co-solvent mixture that allows the dissolution of a dose-appropriate amount of PZQ. Thus, two co-solvent mixtures containing PEG 400, PPG, and water (System 1) and PEG 400, N-methyl-2-pyrrolidone (NMP), and water (System 2) were prepared. The palatability of the optimized PZQ formulation was evaluated in healthy human volunteers, using strawberry and mint as flavors and sucralose as a sweetener. The final formulation was evaluated in terms of solvent intake, drug content, chemical, and physical stability under different stress conditions, and microbiological content. According to the literature, it is the first attempt to prepare PZQ palatable solutions using NMP, PEG 400, and PPG as co-solvent mixtures.

## 2. Materials and Methods

### 2.1. Materials

PZQ (lot PE13604) was purchased from Unifarma (Buenos Aires, Argentina). PEG 400 (lot 14042017) and PPG (lot 14442016) were purchased from Biopack (Buenos Aires, Argentina). NMP (Pharmasolve™, lot 05600168383) was purchased from Ashland (Texas City, TX, USA). Sodium dihydrogen phosphate (Biopack, lot 11585, 99.60% purity), sodium hydrogen phosphate (Biopack, lot 21100, 99.60% purity), methylparaben sodium salt (Novalquim SRL lot 23585, 99.55% purity), mint flavor (Parafarm, lot 49134, 99.30% purity), strawberry flavor (Parafarm, lot 45616, 99.40% purity), and sucralose (Parafarm, lot 41349, 99.55% purity) were purchased from Saporiti (Buenos Aires, Argentina). Water used herein was ultrapure MilliQ water filtered through a pore size of 0.22 μm. United State Pharmacopeia (USP) standards: USP Praziquantel RS, USP Praziquantel Related Compound A RS (2-benzoyl-1,2,3,6,7,11b-hexahydro-4H-pyrazino [2,1-a]isoquinolin-4-one), USP Praziquantel Related Compound B RS (2-(cyclohexyl carbonyl)-2,3,6,7-tetrahydro-4H-pyrazino [2,1-a]isoquinolin-4-one) and USP Praziquantel Related Compound C RS (2-(N-formylhexahydrohippuroyl)-1,2,3,4-tetrahydroisoquinolin-1-one) was obtained from the United States Pharmacopeial Convention (Rockville, MD, USA). Acetonitrile HPLC-grade was supplied by J.T. Baker (Madrid, Spain). All the other reagents and solvents used in this study were of analytical grade.

### 2.2. Design of Experiments

A simplex-centroid mixture design was selected to optimize two different co-solvent systems. Independent factors studied were PEG, PPG, and water for co-solvent System 1 and PEG, NMP, and water for co-solvent System 2. In both cases, factor levels varied between 0 and 1, where 0 corresponded to the lowest value and 1 to the highest value (0 and 100%, respectively), and PZQ solubility (mg/mL) was studied as the response. Design Expert 7 (Stat-Ease, Inc., Minneapolis, MN, USA) was used to generate two randomized designs with 14 experiments each (Table 1). The constraints applied for the optimization process were to maximize water percentage and PZQ concentration as priorities, while lowering the percentage of PPG or NMP.

### 2.3. Drug Solubility Assay

Briefly, an excess amount of PZQ was weighted in vials and 10 mL of each co-solvent mixture (Table 1, *n* = 3) was added and sonicated for 10 min (Ultrasonic cleaner PS-10A, ARCANO, Shandong, China). All vials were shaken for 72 h at 180 rpm at room temperature in an orbital shaker (Boeco OS-20, Hamburg, Germany). After equilibration (4 h), aliquots were filtered with a 0.45 μm membrane filter, diluted with ethanol, and spectrophotometrically measured at 264 nm (Shimadzu UV-1800, Duisburg, Germany). Blank systems, without PZQ, were evaluated spectrophotometrically as a control.

### 2.4. Taste Masking Assay

In agreement with the results obtained in the optimization process, the PZQ formulations (Table 2) were prepared immediately before the assay and provided in transparent glass bottles. Strawberry and mint flavors and sucralose as a sweetener were selected for reducing the perceived bitter taste of PZQ. The assay was performed in a group of 5 healthy volunteers that were asked not to consume any beverage or food 60 min before this study. They were asked to hold 3 mL of each sample formulation in their mouths for 20 s, spat, rinsed with water, and rate the initial taste (IT) of the PZQ solutions on a numerical scale from 0 (not bitter) to 6 (very bitter). Using the same scale, the aftertaste (AT) was rated at 5 min after each trial solution had been spat out to evaluate the residual bitterness of the solutions. In addition, the metallic residual taste was also evaluated after 5 min of spitting up the trial formulations and rated on a numerical scale from 0 (not metallic taste) to 6 (very metallic taste). The protocol of this assay was reviewed and approved by the Bioethics Committee of the National University of Rosario (Argentina) according to the Declaration of Helsinki. Written informed consent was obtained from all participants before enrolment. All statistical tests employed a level of significance of 0.05.

### 2.5. Preparation of PZQ Liquid Dosage Form

A batch of the definitive PZQ dosage form was prepared and poured into separate vials with an elastomeric stopper and the aluminum seal, as detailed in Table 3. PZQ (400 mg) was dissolved in 6.6 mL of NMP and 1.6 mL of PEG 400. Methyl paraben sodium salt (10 mg) was solubilized in distilled water at 90 °C and cooled down after dissolution. Then, 20 μL of mint flavor and 30 mg of sucralose were added as taste-masking excipients. Monobasic sodium phosphate and dibasic sodium phosphate were added to adjust the formulation to pH 7. This aqueous solution was added to the co-solvent mixtures and homogenized under magnetic stirring for 15 min at 300 rpm (room temperature).

### 2.6. Stability Studies

A physical, chemical, and microbiological stability study of a selected final formulation of PZQ was performed. Storage conditions were the following: refrigerated (4 °C, protected from light), room temperature (25 °C, protected and non-protected from light), and high temperature (40 °C, protected from light). On days 30, 60, 90, and 365, three vials were removed from each storage condition, and measurements or evaluations were performed. Color and opalescent aspects were determined by visual inspection performed in a transparent glass vial against a white background. The pH of each sample was measured with a previously calibrated pH meter (Metrohm 744-pH meter, Switzerland). Degradation of PZQ was controlled by high-performance liquid chromatography (HPLC) at the beginning of the assay and on the final day, following the methodology described in Section 2.7

A microbiological assay was performed according to the monograph for non-sterile products of the European Pharmacopoeia. The total aerobic microbial count (TAMC) and the total combined yeast/mold count (TYMC) are 103 and 102 CFU/mL, respectively, and the absence of *Escherichia coli*. Casein soya bean digest broth was inoculated with 1 mL sample, and incubated at 35–37 °C for 18–48 h, and 1 mL of homogenized broth was transferred to 100 mL of MacConkey broth, incubated at 43–45 °C for 18–24 h and on plates of MacConkey Agar at 35–37 °C for 18–72 h.

### 2.7. HPLC Analysis

*Chromatographic system:* The HPLC system consisted of an Agilent HPLC series 1200 (Agilent Technologies, Germany) consisting of a solvent pump (model G1311A), autosampler (model G1329A), column compartment (model G1316A) and DAD (diode-array detector) (model G1315D). Each determination was carried out at 30 °C on a Soroma C18 column (250 × 4.6 mm, 10 μm) and using a mobile phase containing acetonitrile and water in a ratio 40:60 V/V pumped at a flow rate of 1.5 mL/min. A volume of 20 μL of samples was injected per run and chromatograms were acquired at λ = 210 nm. Chromatographic analysis was performed using Ezchrom Elite software, Agilent Technologies.

*Preparation of standard solutions:* PZQ standard solution was prepared by dissolving 20 mg of PZQ in a 100 mL volumetric flask by using a mobile phase. The final concentration obtained was 200 µg/mL. PZQ impurities standard solutions were prepared by dissolving 4 mg of each related compound (A, B, and C) with mobile phase in a 100 mL volumetric flask. The final concentration obtained was 40 µg/mL of each related compound.

*Preparation of test sample:* Samples were diluted to a nominal concentration of 200 µg/mL and filtered through a 0.45 µm nylon syringe filter.

### 2.8. Statistical Analysis

Statistical analysis was performed using GraphPad Prism 4.0 software (GraphPad Software, USA). The results were evaluated by the ANOVA test followed by the Tukey post-test. The results were considered to be significant at a probability level of < 5% (*p* < 0.05).

## 3. Results and Discussions

As reported, a suitable pediatric dosage form for the oral delivery of PZQ is still urgently required [17]. In this regard, the precise selection of excipients used in specific age-adapted oral dosage forms is key regarding safety and acceptable organoleptic properties [29]. Even though PZQ is very soluble in ethanol (97 mg/mL) [30], the American Academy of Pediatrics (AAP) and other reports do not recommend it in pediatric formulations due to the lack of information regarding its effects on children [31,32]. Thus, in this work, ethanol was avoided and replaced by other solvents such as PEG 400, PPG, and NMP [33,34].

### 3.1. Drug Solubility Assay

In agreement with previous experiments performed in our lab [35], we wondered whether different combinations of water-miscible solvents, such as PEG 400, PPG, and NMP, could solubilize PZQ. Thus, two systems containing PEG 400, PPG, and water and NMP, PEG 400, and water were prepared for solubilizing PZQ. The solubility of PZQ in water was 0.38 mg/mL, while NMP exhibited a higher solubilization capacity (146 mg/mL) than PEG 400 (17 mg/mL) and PPG (12 mg/mL). These results correlate with the differences in the polarity of the solvents. NMP is a non-ionic solvent miscible with water showing a great capacity to improve the solubility and permeability of hydrophobic drugs, either as NMP/water or NMP/co-solvent mixtures [36,37,38,39,40,41]. It is less polar than PEG 400 and PPG and may be capable of further reducing the interfacial tension with the non-polar PZQ, leading to higher solubilization. PEG is less hydrophilic than PPG, which could lead to higher interaction with the drug [42,43]. Since solvent mixtures may further increase the drug solubility, a simplex centroid mixture design was used to study whether those mixtures would impact the PZQ solubility. PEG 400, PPG, and water (System 1) and NMP, PEG 400, and water (System 2) were the independent variables, and the response measured was the concentration of PZQ (mg/mL) (Table 1). The standard errors in both designs were <0.7, which indicated the suitability and acceptable predictions of the design. Different models (linear, quadratic, and special cubic) were tested for goodness of fit. Quadratic models were chosen for modeling the response in both co-solvent systems because showed a non-significant lack of fit, and R-squares were in good agreement with predicted- R-squares. The calculated F values for the quadratic model were large, 85.20 and 161.37 for System 1 and 2, respectively, and the values of residual error indicated that the variation in PZQ solubilization can be explained by the chosen model (Table 4). All the combinations of the solvent used presented a statistical significance (*p*-value < 0.05), except for NMP and water.

**Figure 1:**
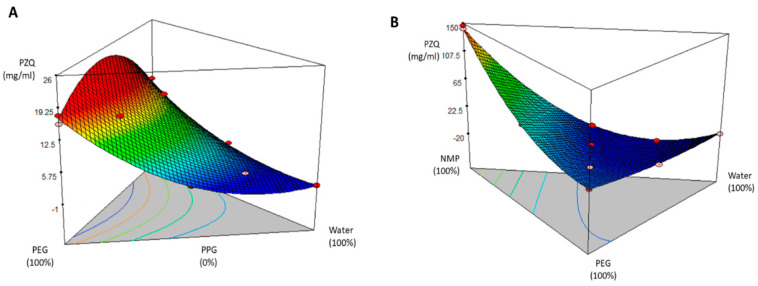
Effect of solvents proportions in PZW solubilization through RSM for co-solvent System 1 (**A**) and co-solvent System 2 (**B**).

The response surface methodology (RSM) is widely used to estimate how the factors (co-solvent ratio) can influence the response (PZQ solubility). Herein, the results obtained from the experiments (Table 1) revealed a direct relationship between the solvent concentrations and PZQ solubility. In the case of System 1 (PEG 400/PPG/water), the drug solubility increased when the proportion of the PEG 400 was increased against the water proportion while keeping the concentration of PPG constant. In the case of System 2 (PEG 400/NMP/water) where the drug solubility kept increasing as the proportion of NMP increased while keeping the concentration of PEG 400 constant, suggesting that the solvent polarity is fundamental in the solubilization of the non-polar PZQ [43,44]. In Figure 1 there is a clear trend of decreasing PZQ concentration, which was expected, as the presence of water, the most polar solvent, increased. As shown in Figure 1A, the findings revealed that the optimum value of PZQ was 14.80 ± 0.24 mg/mL, which corresponds to the co-solvent system PEG 400 (67%), PPG (16.5%), and W (16.5%), while the system containing PEG 400 (16.5%), PPG (67%), and water (16.5%) solubilized 12.25 ± 0.33 mg/mL of the drug. 

It is important to note that the system PEG 400 (33.3%), PPG (33.3%), and water (33.3%) solubilized only 7.64 ± 0.62 mg/mL of the drug, suggesting that the presence of water decreased to a large extent the solubility of PZQ in comparison with the other two assayed systems. Regarding System 2, the optimum amount of PZQ in solution was 146.69 ± 5.48 mg/mL in NMP (100%). However, an oral solution based only on NMP would exhibit several potential toxic issues and difficulties regarding palatability and patient compliance. A closer inspection of Table 1 shows that the NMP-PEG 400 mixture (50–50%) had a PZQ concentration of 50.04 ± 4.58 mg/mL, while the counterpart, the PPG-PEG 400 mixture with the same proportions, had solubilized only 7.50 ± 0.19 mg/mL. From all the data presented in Table 1, it is evident that NMP acted as a better solubilizing agent. The higher solubilization power of NMP in different mixtures can be explained by the dual mechanism that NMP uses in solubilization processes as a co-solvent and complexing agent [44]. The ability to solubilize poorly water-soluble molecules using mixtures of water and organic solvents leads to a lower polar environment and higher solvation of such molecules in the aqueous phase [45]. Based on the data, an optimization process was performed, looking for higher PZQ solubilization and prioritizing the water content in the mixtures. The optimized co-solvent mixtures were 67% PEG 400, 16.5% PPG, and 16.5% water for System 1, solubilizing 14 mg/mL of PZQ, and 67% NMP, 16.5% PEG 400, and 16.5% water for cosolvent System 2, which solubilized 44.36 ± 3.74 mg/mL and it could be considered as a more suitable solution for developing a final product.

### 3.2. Solvents Daily Intake

As described, one of the main issues related to organic solvents as vehicles of pharmaceutical products is their general toxicity, tissue damage, and the modification of cellular functions [33]. Regarding the prescription of PZQ, a single dose of 5 mg/kg gives a cure rate of nearly 90% in patients infected with *T. saginata* and *T. solium* [46]. Given these facts, it is necessary to evaluate the daily intake of each solvent for the optimized solutions. Thus, the daily intakes of PEG 400/PPG and PEG 400/NMP are shown in Table 5A,B, according to children’s age and body weight.

PEG 400 exhibits a low toxicity profile [47]. It is not degraded in the intestinal tract, not metabolized, and excreted in the urine. The Food and Drug Administration Inactive Ingredient database indicated that the maximum amount of PEG 400 permitted for use in approved drug products for oral administration is 960.48 mg per dosage form, while the Japanese Pharmaceutical Excipients Directory specifies a highest maximum permitted amount of 2.31 g/day of PEG 400 by oral route [48]. Considering these limits, the maximum daily intake of PEG 400 would be nearly 0.26 g/kg for cosolvent system 1 and 0.35 g/kg for cosolvent system 2. Both values are far away from the reported maximum allowed concentration (2.31 g/day).

Regarding PPG, it is used in several commercially available formulations, usually up to 1000 mg/kg [25,49]. However, it is not recommended for patients below four years due to the significant limitations concerning its metabolism, which may lead to the accumulation of PPG in the systemic circulation and tissues [50]. In this respect, for co-solvent system 1, the maximum daily intake of PPG is up to 0.28 g/kg, suggesting that it could not exhibit toxicity issues.

In the case of co-solvent system 2, NMP shows low toxicity by oral and parenteral routes of administration, and it is being used as a solvent for different pharmaceutical products [51]. According to the literature, NMP is a Category 5 (LD50 between 2000 and 5000 mg/kg body weight) substance which suggests a relatively low acute toxicity hazard but might show a danger to vulnerable populations [52]. In connection with it, a toxicity study of an NMP-based self-nano emulsifying drug delivery system was performed in mice for seven days. The results suggested that such a formulation with 10 mg/kg of NMP did not significantly alter the evaluated parameters of the animal model [53]. Moreover, it is worth noting that there are two commercially available medicines formulated with high concentrations of NMP (leuprolide acetate solution with 55% to 66% [53], and ATRIGEL^®^ with 63.3% [44]). As seen in Table 5B, the maximum daily intake of NMP was up to 0.07 mL/kg (0.076 g/kg), which might have some concerns regarding potential toxic effects. To elucidate it, a detailed safety profile is needed to confirm the suitability of the solutions based on NMP-PEG 400 co-solvents. However, it is worth mentioning that PZQ is prescribed as a single dose for the therapy of *T. solium* and *T. saginata*, and the patients would ingest once such solvent mixture.

### 3.3. Taste Masking Assay

The palatability of a pharmaceutical formulation refers to how pleasant or acceptable it can be in terms of taste, smell, and overall sensory experience when administered to patients. It is a critical factor in medication adherence, especially for children and those with swallowing difficulties or sensory sensitivities. PZQ exhibits an unpleasant taste [22], which is challenging to mask, making the formulations less palatable. The chosen optimized co-solvent mixture with NMP, PEG 400, and water was prepared with a final PZQ concentration of 40 mg/mL, adding different flavors and a sweetener to evaluate the better combination for taste masking (Table 2). Typical flavors include cherry, orange, strawberry, and mint, while common sweeteners include sucrose, glucose, and artificial sweeteners. However, caution must be taken with sweeteners, particularly in diabetic patients or those with dietary restrictions.

**Figure 2:**
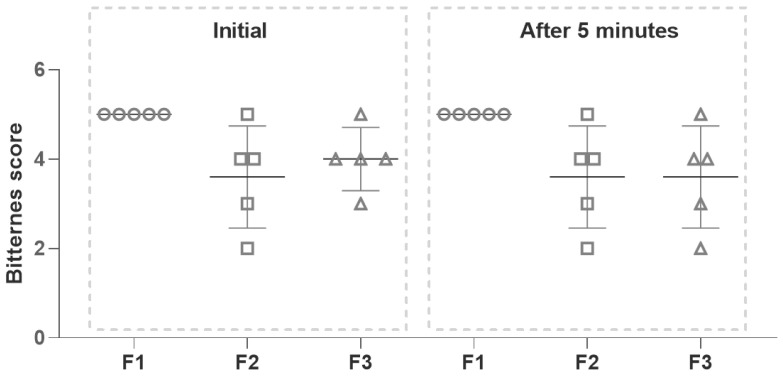
Bitterness scores of PZQ formulations (initial taste I_T_ and after taste A_T_).

Thus, for reducing PZQ bitterness, strawberry and mint flavors were evaluated as potential taste masking [54]. Sucralose, a calorie-free artificial substance nearly 600 times sweeter than sucrose and three times sweeter than aspartame, was selected as a sweetener due to its stability, safety, and palatability [55]. The bitterness intensity of PZQ solutions was determined in a randomized assay under the same conditions using a panel of human volunteers in an intake and spit test [56]. The tested formulations were not swallowed. The bitterness level of the optimized final formulation containing strawberry and sucralose (F2) or mint and sucralose (F3) was rated and compared with a solution of PZQ without flavors and sweetener (F1). As seen in Figure 2, the IT assay of the F2 solution indicated that one volunteer scored a value of 5, two volunteers scored a value of 4, one volunteer scored a value of 3, and the other one a value of 2. Concerning the F3, the IT showed that one volunteer scored a value of 5, three volunteers scored a value of 4, and one volunteer scored a value of 3. In the case of the AT assay, the performance of F2 was identical to the IT assay, and the evaluation of F3 showed a slight modification because one volunteer scored a value of 5, two volunteers scored a value of 4, one volunteer scored a value of 3 and the other one a value of 2. These results suggested that both strawberry and mint flavors would be effective additives to reduce the bitterness of PZQ to some extent (25%–75%). 

**Figure 3:**
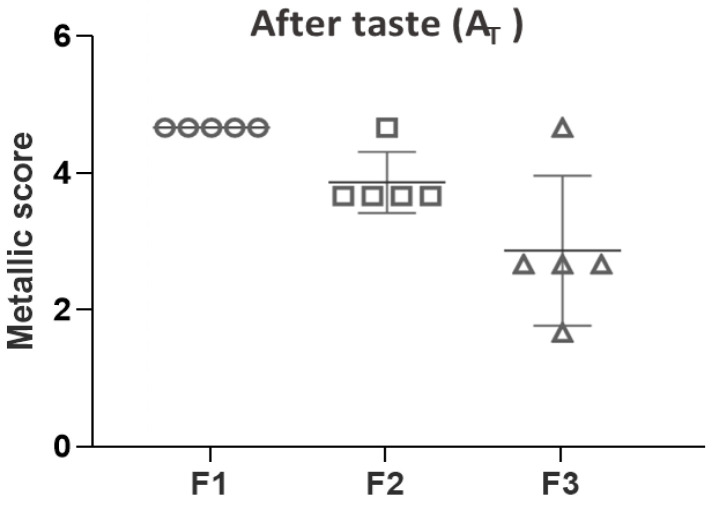
Metallic scores of PZQ formulations (after taste A_T_).

As seen in Figure 3, the evaluation of metallic residual taste indicated that one volunteer scored a value of 5 and the other four scored a value of 4 for the strawberry flavored solution while one volunteer scored a value of 5, three volunteers scored a value of 3 and the other one a value of 2 for the mint-flavored solution. Interestingly, such differences in favor of the mint-flavored solution would be related to the higher capacity of mint to mask the metallic taste [57,58]. Regarding both palatability assays, it could be more convenient to mask the unpleasant PZQ taste using mint as flavor and sucralose as sweetener, even though fruit flavors are preferred by pediatric patients in some circumstances [59]. It is worth mentioning that such a combination of excipients was successfully used in a pilot study with fifteen human volunteers to reduce the bitter taste of microencapsulated quinine HCl [60].

### 3.4. Stability Studies

**Figure 4:**
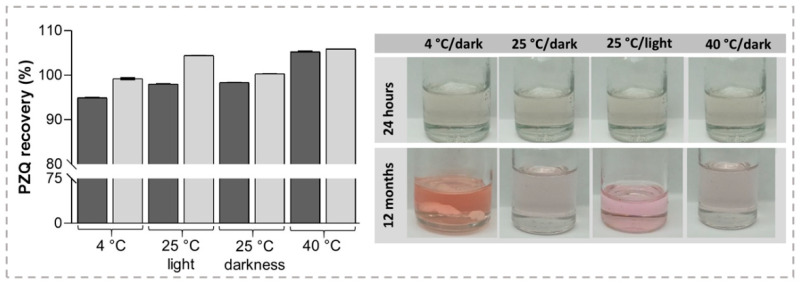
The recovery percentage of PZQ, initial and after 12 months, alongside pictures showing the color changes presented by samples.

The long-term stability of liquid formulations represents one of the main issues since drug precipitation is a very feasible problem, alongside with degradation of the active ingredient. Herein, the effect of pH, storage temperature, and light on the stability of PZQ was evaluated. According to these findings, the pH of the PZQ-optimized NMP/PEG 400/W solution was kept constant during the considered time in all conditions studied. In this regard, United State Pharmacopeia (USP) has identified three impurities related to PZQ: impurity A, impurity B, and impurity C. Regardless that PZQ concentration shows little or no change over time within the acceptance criteria for that attribute, as it is defined by different pharmacopeias for oral solution dosage forms (90.0% ≤ labeled amount ≤ 110.0%), the presence of its pharmacopeia impurities was observed after diverse storage conditions. In samples stored at 40 °C non-significant differences were found in recovery percentage after storage (*p* = 0.1354) (Figure 4). In the other evaluated storage conditions, recovery percentages were higher than expected (*p* = 0.0023 for 4 °C and *p* = 0.0001 for 25 °C with/without light exposure).

Even though PZQ concentration remain inside an acceptable concentration range, a color change was observed during storage. As seen in Figure 4, samples stored at 25 °C and protected from light kept their initial transparency and clarity for up to 60 days, while the solutions kept at 25 °C and non-protected from the light had a more intense pink color. Samples stored at 40 °C displayed similar behavior in terms of color to those kept at 25 °C and protected from light. Under these conditions, no precipitate was observed revealing that the co-solvent mixture may keep the drug in solution, even up to 365 days. On the contrary, the stability of the samples stored at 4 °C showed some concern regarding the concentration and transparency. The colorless solution of PZQ became pink after being kept in the refrigerator. In addition, it was also observed the presence of a white precipitate after 60 days, which remained for 365 days.

**Figure 5:**
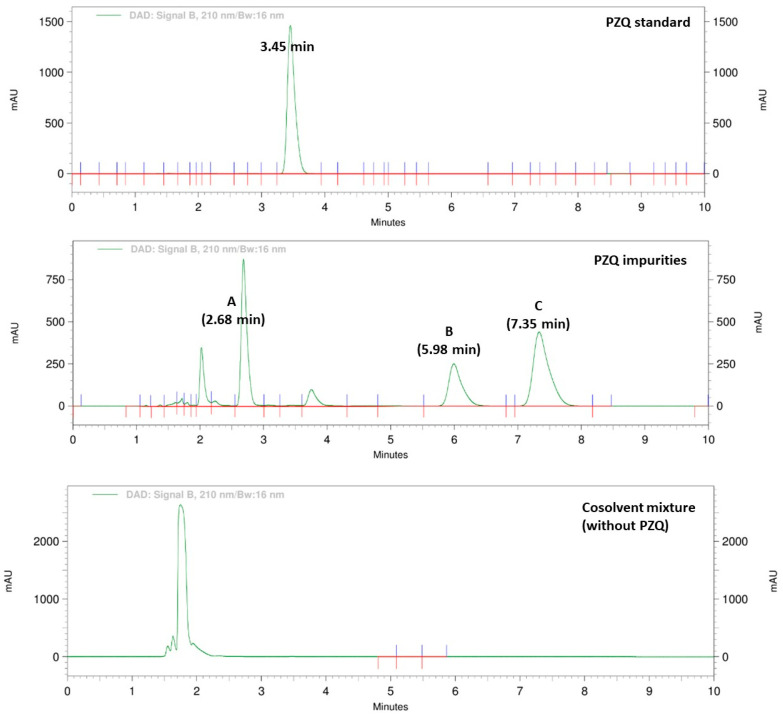
Chromatograms of pure PZQ, the mixture of PZQ USP impurities, and the cosolvent mixture (without PZQ).

As expected, lower temperature leads to a decrease of the drug solubility leading to the precipitation of, probably, PZQ. To elucidate it, such precipitate was filtered, dried at 40 °C for 48 h, and analyzed by differential scanning calorimetry (DSC). This study showed a single endothermic signal at 137.6 °C (Figure S1), which is in accord with the reported melting point of PZQ [15,19]. Thus, the precipitation of the drug from the co-solvent solutions was seen when stored at low temperatures after 60 days. Regarding the development of pink color in PZQ formulation, such a phenomenon was already reported during the preparation of different solid systems of the drug [61,62,63]. Then, chromatographic runs of individual and mixed PZQ related compounds were performed. As shown in Figure 5, PZQ related compound chromatograms present three major peaks with retention times (Tr) of 2.70, 6.00 min, and 7.40 min corresponding to PZQ related compounds A, B, and C, respectively. 

**Figure 6:**
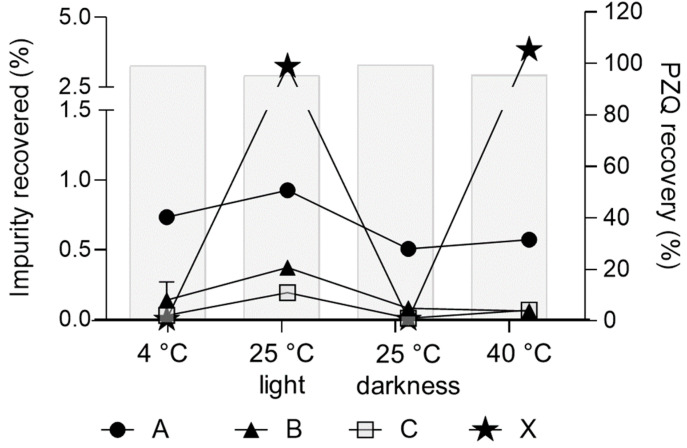
Impurities generated at different storage conditions over a period of 12 months (*y-left* axis). The recovery% of PZQ is shown as light-grey bars (*y-right* axis).

Initially, in chromatograms of the developed liquid formulation, only two peaks were observed, and they corresponded to PZQ (3.45 min) and the co-solvent mixture (1.74 min). After 24 h, no changes were observed on chromatograms of the samples stored at 4 °C, 25 °C with and without light exposure, and at 40 °C, since only signals attributed to pure PZQ and co-solvent mixture were observed (Table S1 and Figure S1). However, after 12 months of storage under the same conditions, all samples showed a new peak with Tr of 2.3 min, with higher intensity on those samples stored at 4 °C, whose simultaneously are the ones that developed a more intense pink coloration. Figure 6 illustrates the generation of different impurities at different conditions. As mentioned, impurity A appeared at higher concentrations, followed by impurity B. In addition, an unknown impurity showed a Tr around 2.10 min and only occurred when the formulation was submitted to light (at 25 °C) and temperature (40 °C). In this regard, it was described [20] the appearance of an impurity during the formulation of PZQ microparticles, which is not defined in the USP. These findings suggest that PZQ suffers physical or chemical modifications regardless of different excipients, solvents, or storage conditions. 

It can be concluded that the optimal conditions for storage of the developed liquid formulation are at room temperature and protected from light, since it was the condition where impurities were underneath the acceptable values, and the recovery percentage of PZQ was over 99%. Even though oral solutions do not need to be sterile, the microbiological stability was also analyzed to evaluate if the preservative (methylparaben sodium salt) would be able to protect the definitive formulation from microbiological growth or from microorganisms that could be introduced during or after the manufacturing process and/or from repeatedly withdrawing doses. The result of this assay indicated that PZQ liquid samples showed good microbiological stability since no microbial growth was observed during storage. The lack of microbiological contamination in this period shows the suitability of the preservative regardless of the conditions of storage. Thus, the requirement of pharmacopeias was provided, and the solution was microbiologically preserved.

## 4. Conclusions

In conclusion, four optimized formulations were successfully obtained through a quality-by-design approach to estimate the maximum drug concentration in the solution. The solubility of PZQ in water is 0.4 mg/mL, which was increased up to 14.80 ± 0.24 mg/mL, in the presence of the co-solvent system PEG 400 (67%), PPG (16.5%), and water (16.5%), while drug solubility increased up to 44.36 ± 3.74 mg/mL when a mixture of NMP (67%), PEG 400 (16.5%), and water (16.5%) was assayed. The palatability of the optimized solution was evaluated in human volunteers in an intake and spit test, and the assay showed that both strawberry and mint flavors reduced the bitterness of PZQ. Regarding the metallic residual taste, the mint-flavored solution was preferred to the strawberry-flavored solution to lower such drawbacks. Considering the limits of solvent intake, the daily intakes of PEG 400 and PPG are far from the reported maximum allowed amounts, while NMP solvent intake might raise some toxicity issues. The stability studies indicated that the optimal conditions for storage of the developed liquid formulation are at room temperature and protected from light because the existing impurities were underneath the acceptable values, and the recovery percentage of PZQ was over 99%. In conclusion, these novel PZQ solutions should be considered for further preclinical and clinical studies considering the high prevalence of this tapeworm infection in children.

## Figures and Tables

**Table 1:** Simplex-centroid mixture design. Independent factors were: PPG, PEG, and water (W) (co-solvent System 1) and NMP, PEG, and W (co-solvent System 2). PZQ solubility (mg/mL) was studied as the response. Responses are presented as average (standard deviation), for *n* = 3.

	Cosolvent System 1					Cosolvent System 2			
	Factors			Response		Factors			Response
	PPG	PEG	W	PZQ (mg/mL)		NMP	PEG	W	PZQ (mg/mL)
1	0.16	0.66	0.16	14.91 (0.24)	15	0.16	0.66	0.16	10.60 (0.17)
2	0.33	0.33	0.33	7.75 (0.42)	16	0.33	0.33	0.33	7.74 (0.32)
3	0.00	1.00	0.00	16.15 (0.14)	17	0.00	1.00	0.00	19.19 (1.69)
4	0.00	1.00	0.00	17.91 (0.27)	18	0.00	1.00	0.00	17.42 (2.12)
5	1.00	0.00	0.00	11.86 (1.06)	19	1.00	0.00	0.00	146.70 (5.49)
6	0.16	0.16	0.66	0.89 (0.01)	20	0.16	0.16	0.66	1.73 (0.14)
7	0.00	0.00	1.00	0.21 (0.02)	21	0.00	0.00	1.00	0.22 (0.00)
8	0.66	0.16	0.16	12.36 (0.33)	22	0.66	0.16	0.16	39.71 (3.74)
9	1.00	0.00	0.00	9.03 (0.38)	23	1.00	0.00	0.00	141.70 (11.29)
10	0.00	0.50	0.50	1.55 (0.05)	24	0.00	0.50	0.50	1.42 (0.14)
11	0.50	0.00	0.50	2.60 (0.01)	25	0.50	0.00	0.50	4.00 (0.44)
12	0.50	0.50	0.00	7.50 (0.19)	26	0.50	0.50	0.00	50.04 (4.58)
13	0.00	0.00	1.00	0.38 (0.02)	27	0.00	0.00	1.00	0.21 (0.02)
14	0.00	0.50	0.50	1.73 (0.05)	28	0.00	0.50	0.50	1.75 (0.05)

**Table 2:** Composition of PZQ formulations for taste masking.

Code Name	Composition
**F1**	PZQ + co-solvent mixture
**F2**	PZQ + co-solvent mixture + 0.2% of strawberry flavor + 30 mg of sucralose
**F3**	PZQ + co-solvent mixture + 0.2% of mint flavor + 30 mg of sucralose

**Table 3:** The definitive oral liquid formulation of PZQ (10 mL).

PZQ	400 mg
PEG 400	1.6 mL
NMP	6.6 mL
Water	1.78 mL
Methyl paraben sodium salt	10 mg
Sucralose	30 mg
Mint flavor	20 μL
Na_2_HPO_4_	66 mg
NaH_2_PO_4_	24 mg

**Table 4:** ANOVA values for quadratic models applied to both co-solvent systems.

	Source	Sum of Squares	Mean Square	F Value	*p*-Value
Cosolvent system 1	Model	514.34	102.87	85.20	<0.0001
	PEG-PPG	33.29	33.29	27.57	0.0012
	PEG-water	9.19	9.19	7.61	0.0281
	PPG-water	71.90	71.90	59.54	0.0001
Cosolvent system 2	Model	32,247.58	6449.52	161.37	<0.0001
	PEG-NMP	1178.95	1178.95	29.50	0.0006
	PEG-water	4790.56	4790.56	119.86	<0.0001
	NMP-water	63.17	63.17	1.58	0.2441

**Table 5:** Daily intakes of PEG 400/PPG (A) and PEG 400/NMP (B) pediatric patients based on quantities present in different formulations.

		1 Year Old (~10 kg)	2 Years Old (~12.5 kg)	4 Years Old (~16 kg)	5 Years Old (~18 kg)	7 Years Old (~23 kg)
A	17% PPG, 66% PEG 400, 17% water					
	Final volume (mL) to be administrated:	3.37	4.22	5.40	6.08	7.77
	mL PEG:	2.26	2.82	3.62	4.07	5.20
	mL PPG:	0.57	0.71	0.91	1.03	1.32
	66% PPG, 17% PEG 400, 17% water					
	Final volume (mL) to be administrated:	4.08	5.10	6.53	7.35	9.39
	mL PEG:	0.70	0.80	1.11	1.25	1.60
	mL PPG:	2.73	3.42	4.37	4.92	6.29

B	17% NMP, 66% PEG 400, 17% water					
	Final volume to be administrated:	4.62	5.77	7.40	8.32	10.63
	mL NMP:	0.76	0.96	1.23	1.38	1.76
	mL PEG:	3.07	3.85	4.90	5.50	7.08
	66% NMP, 17% PEG 400, 17% water					
	Final volume to be administrated:	1.13	1.41	1.80	2.03	2.60
	mL NMP:	0.75	0.94	1.20	1.36	1.73
	mL PEG:	0.19	0,24	0.30	0.34	0.44

## Data Availability

No new data were created or analyzed in this study. Data sharing is not applicable to this article.

## Supplementary Materials

The following supporting information can be downloaded at: https://www.mdpi.com/article/10.3390/pharmaceutics15082050/s1, Figure S1: DSC from precipitated PZQ; Figure S2: Chromatogram for samples stored at 4 °C for 12 months. The peak at 1.739 min from cosolvent mixture, 2.337 min from impurity A, and at 3.444 min of PZQ; Figure S3: Chromatogram for samples stored at 25 °C and under darkness conditions for 24 h. The peak at 1.738 min from cosolvent mixture and the one at 3.443 min of PZQ; Figure S4: Chromatogram for samples stored at 25 °C and under darkness conditions for 12 months. The peak at 1.740 min from the cosolvent mixture and the one at 3.447 min from PZQ. A low signal from impurity A can be seen at 2.338; Figure S5: Chromatogram for samples stored at 25 °C under light exposure for 24 h for. The peak at 1.737 min from the cosolvent mixture and the one at 3.441 min from PZQ; Figure S6: Chromatogram for samples stored at 25 °C under light exposure for 12 months. Peak at 1.739 min from cosolvent mixture, at 2.337 min from impurity A and the one at 3.443 min from PZQ; Figure S7: Chromatogram for samples stored at 40 °C for 24 h. The peak at 1.745 min from cosolvent mixture and the one at 3.446 min from PZQ; Figure S8: Chromatogram for samples stored at 40 °C for 12 months. The peak at 1.741 min from cosolvent mixture and the one at 3.439 min from PZQ; Table S1: Chromatographic information of samples at the beginning and after concluding the assay; Table S2: Chromatographic information (retention time and peak area and height) for samples stored at 4 °C for 12 months; Table S3: Chromatographic information (retention time and peak area and height) for samples stored at 25 °C and under darkness conditions for 24 h; Table S4: Chromatographic information (retention time and peak area and height) for samples stored at 25 °C and under darkness conditions for 12 months; Table S5: Chromatographic information (retention time and peak area and height) for samples stored at 25 °C under light exposure for 24 h; Table S6: Chromatographic information (retention time and peak area and height) for samples stored at 25 °C under light exposure for 12 months; Table S7: Chromatographic information (retention time and peak area and height) for samples stored at 40 °C for 24 h; Table S8: Chromatographic information (retention time and peak area and height) for samples stored at 40 °C for 12 months.
