# Supplementary material for: Preformulation and Long-Term Stability Studies of an Optimized Palatable Praziquantel Ethanol-Free Solution for Pediatric Delivery
Source: Pharmaceutics. 2023 Jul 30;15(8):2050. doi: 10.3390/pharmaceutics15082050 (PMC10458622; doi:10.3390/pharmaceutics15082050)

Supplementary information: HPLC runs

Table S1: Chromatographic information (retention time and peak area and height) for samples stored at 4 °C for 24 hours.

DAD: Signal B,
210 nm/Bw:16 nm
Results

Retention Time	Area	Area %	Height	Height %
0.047	933	0.00	165	0.00
0.195	702	0.00	118	0.00
0.313	655	0.00	131	0.00
0.693	8149	0.01	442	0.01
0.976	8774	0.01	1386	0.02
1.243	495637	0.69	79352	0.92
1.431	31167	0.04	5319	0.06
1.625	85147	0.12	20440	0.24
1.738	45927798	63.71	5540357	64.32
2.332	342889	0.48	36849	0.43
2.669	81470	0.11	9080	0.11
2.843	139208	0.19	9218	0.11
3.438	24899843	34.54	2906410	33.74
4.013	17261	0.02	1460	0.02
4.337	25427	0.04	1174	0.01
4.858	8888	0.01	499	0.01
6.015	12544	0.02	808	0.01
Totals	72086492	100.00	8613208	100.00

Figure S1: Chromatogram for samples stored at 4 °C for 24 hours. The peak at 1.738 min from cosolvent mixture and the one at 3.438 min of PZQ

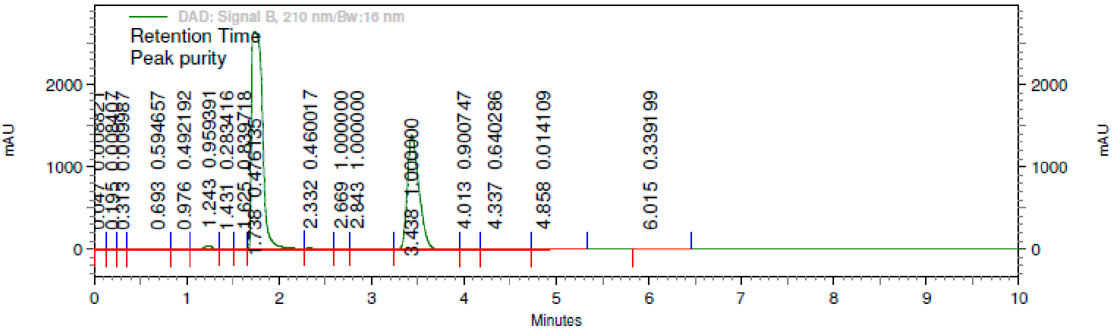

Table S2: Chromatographic information (retention time and peak area and height) for samples stored at 4 °C for 12 months

DAD: Signal B, 210 nm/Bw:16 nm
Results

Retention Time	Area	Area %	Height	Height %
0.040	1034	0.00	135	0.00
0.268	335	0.00	62	0.00
0.412	131	0.00	42	0.00
0.650	885	0.00	89	0.00
1.492	8764935	9.43	1227871	10.18
1.544	1155782	1.24	325553	2.70
1.627	1545467	1.66	420837	3.49
1.739	46757509	50.33	5565340	46.16
2.010	1128684	1.21	144283	1.20
2.337	7089441	7.63	1309852	10.86
2.651	189484	0.20	20089	0.17
2.843	86265	0.09	9731	0.08
3.073	77880	0.08	6043	0.05
3.444	26032556	28.02	3021914	25.06
4.033	17396	0.02	1329	0.01
4.334	22561	0.02	1124	0.01
4.870	5095	0.01	386	0.00
5.068	735	0.00	211	0.00
5.178	2296	0.00	206	0.00
5.500	1407	0.00	65	0.00
5.779	359	0.00	111	0.00
6.027	11785	0.01	606	0.01
7.177	80	0.00	32	0.00
7.398	5324	0.01	262	0.00
8.091	603	0.00	54	0.00
8.291	144	0.00	51	0.00
8.360	172	0.00	46	0.00
9.176	399	0.00	62	0.00
9.252	378	0.00	57	0.00
**Totals**	92899122	100.00	12056443	100.00

Figure S2: Chromatogram for samples stored at 4 °C for 12 months. The peak at 1.739 min from cosolvent mixture, 2.337 min from impurity A, and at 3.444 min of PZQ

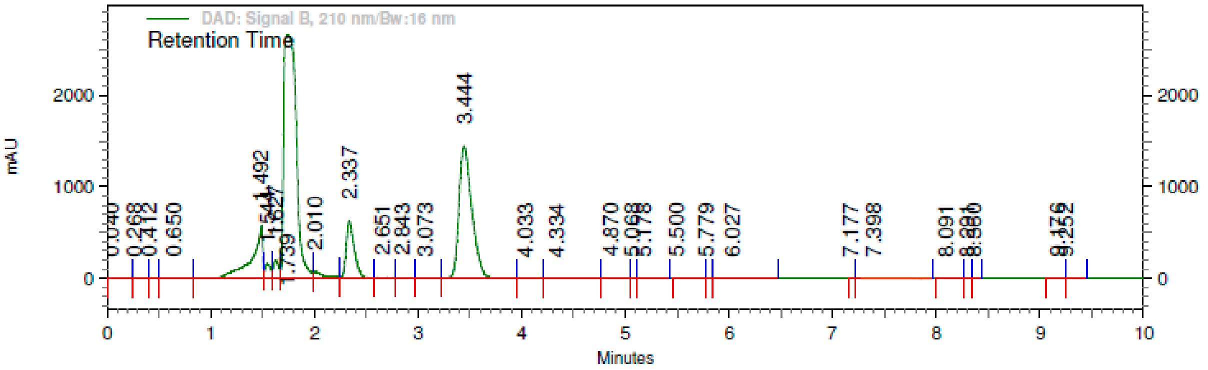

Table S3: Chromatographic information (retention time and peak area and height) for samples stored at 25 °C and under darkness conditions for 24 hours

DAD: Signal B, 210 nm/Bw:16 nm Results

Retention Time	Area	Area %	Height	Height %
0.048	1332	0.00	200	0.00
0.154	915	0.00	166	0.00
0.302	2596	0.00	246	0.00
0.968	16886	0.02	1330	0.02
1.238	522032	0.71	86272	0.99
1.433	40948	0.06	6392	0.07
1.625	95478	0.13	22371	0.26
1.738	46357447	62.98	5546949	63.50
2.334	367609	0.50	38640	0.44
2.672	81771	0.11	9756	0.11
2.849	168216	0.23	11697	0.13
3.443	25809734	35.07	3004913	34.40
4.023	25586	0.03	1961	0.02
4.311	41699	0.06	1731	0.02
4.866	30223	0.04	1046	0.01
5.423	8552	0.01	519	0.01
6.013	29812	0.04	1038	0.01
6.751	276	0.00	128	0.00
6.815	1512	0.00	154	0.00
7.388	232	0.00	55	0.00
9.028	546	0.00	52	0.00
9.267	272	0.00	38	0.00
Totals	73603674	100.00	8735654	100.00

Figure S3: Chromatogram for samples stored at 25 °C and under darkness conditions for 24 hours. The peak at 1.738 min from cosolvent mixture and the one at 3.443 min of PZQ

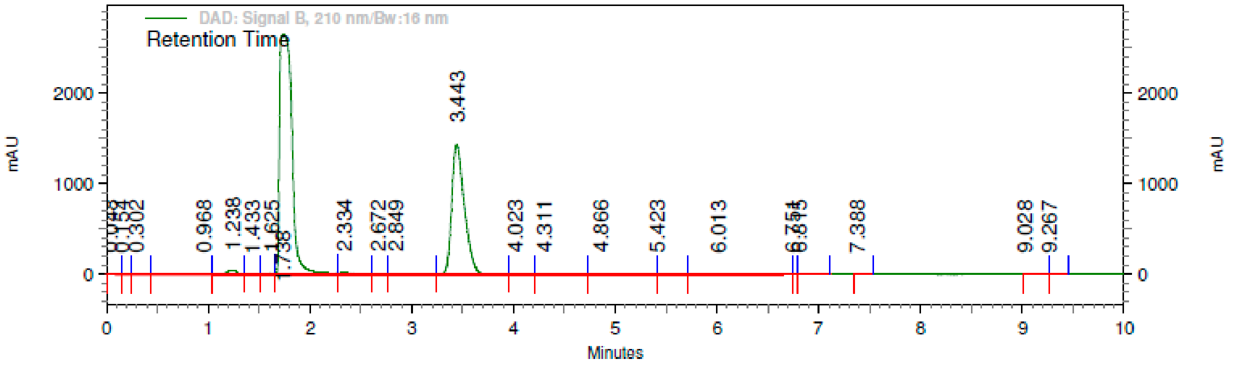

Table S4: Chromatographic information (retention time and peak area and height) for samples stored at 25 °C and under darkness conditions for 12 months

DAD: Signal B, 210 nm/Bw:16 nm

Results

Retention Time	Area	Area %	Height	Height %
0.038	769	0.00	143	0.00
0.198	571	0.00	131	0.00
0.283	1410	0.00	172	0.00
0.735	13086	0.02	716	0.01
1.313	2680313	3.32	229179	2.38
1.546	1288868	1.60	333052	3.46
1.628	891158	1.10	248615	2.58
1.740	48216445	59.69	5570319	57.91
2.338	978404	1.21	153598	1.60
2.659	137974	0.17	13200	0.14
2.835	66662	0.08	7512	0.08
2.996	66910	0.08	5006	0.05
3.447	26329661	32.60	3051652	31.73
4.049	18108	0.02	1451	0.02
4.343	32271	0.04	1234	0.01
4.868	13590	0.02	718	0.01
5.405	193	0.00	54	0.00
6.032	24220	0.03	1240	0.01
6.796	9780	0.01	303	0.00
7.427	2486	0.00	167	0.00
7.761	454	0.00	42	0.00
8.011	85	0.00	54	0.00
8.044	124	0.00	26	0.00
Totals	80773542	100.00	9618584	100.00

Figure S4: Chromatogram for samples stored at 25 °C and under darkness conditions for 12 months. The peak at 1.740 min from the cosolvent mixture and the one at 3.447 min from PZQ. A low signal from impurity A can be seen at 2.338.

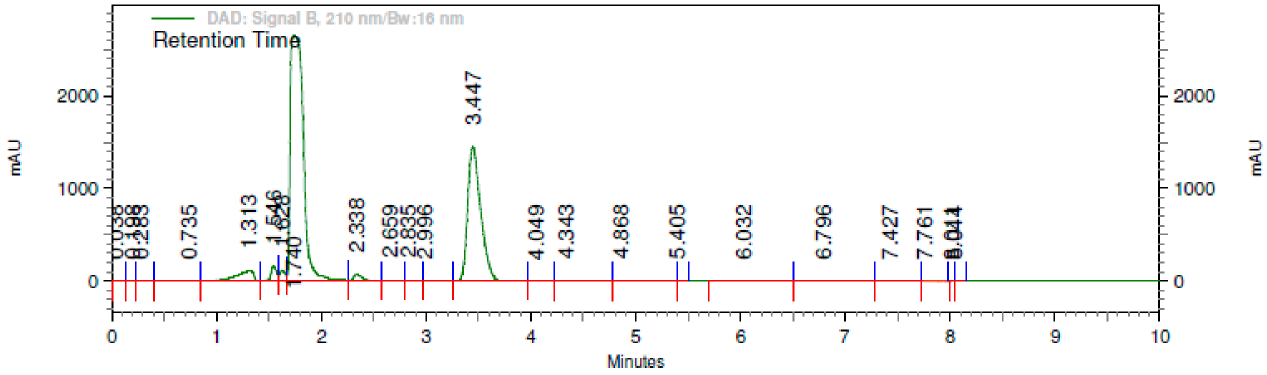

Table S5: Chromatographic information (retention time and peak area and height) for samples stored at 25 °C under light exposure for 24 hours

DAD: Signal B, 210 nm/Bw:16 nm
Results

Retention Time	Area	Area %	Height	Height %
0.044	1250	0.00	166	0.00
1.162	476746	0.65	58040	0.67
1.423	21014	0.03	5729	0.07
1.473	14172	0.02	4041	0.05
1.629	117235	0.16	30916	0.36
1.737	46221789	63.09	5551997	63.92
2.333	357689	0.49	38195	0.44
2.673	77199	0.11	9161	0.11
2.847	169618	0.23	12748	0.15
3.441	25720339	35.10	2969976	34.19
4.037	21927	0.03	1604	0.02
4.356	40007	0.05	1511	0.02
4.877	18128	0.02	945	0.01
6.004	10050	0.01	563	0.01
Totals	73267163	100.00	8685592	100.00

Figure S5: Chromatogram for samples stored at 25 °C under light exposure for 24 hours for. The peak at 1.737 min from the cosolvent mixture and the one at 3.441 min from PZQ.

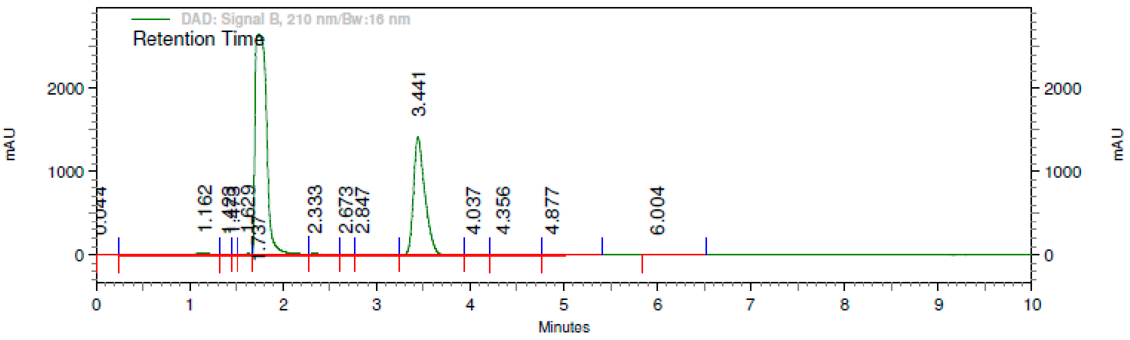

Table S6: Chromatographic information (retention time and peak area and height) for samples stored at 25 °C under light exposure for 12 months

DAD: Signal B, 210 nm/Bw:16 nm
Results

Retention Time	Area	Area %	Height	Height %
0.023	926	0.00	111	0.00
0.287	985	0.00	86	0.00
0.790	113	0.00	54	0.00
1.117	115719	0.14	17776	0.16
1.285	17433	0.02	3446	0.03
1.542	1378172	1.61	344550	3.19
1.626	1823919	2.14	493579	4.57
1.739	47150404	55.22	5548180	51.33
2.140	933650	1.09	140649	1.30
2.337	5640410	6.61	1026386	9.50
2.651	270644	0.32	32095	0.30
2.849	275131	0.32	24445	0.23
3.443	27412430	32.10	3156869	29.21
4.030	26977	0.03	2308	0.02
4.311	44502	0.05	2590	0.02
4.699	19474	0.02	1674	0.02
4.977	69488	0.08	2773	0.03
5.586	17531	0.02	980	0.01
6.043	116657	0.14	6124	0.06
6.584	5013	0.01	431	0.00
6.871	8470	0.01	476	0.00
7.438	60595	0.07	2791	0.03
9.448	4032	0.00	182	0.00
9.937	102	0.00	37	0.00
Totals	85392777	100.00	10808592	100.00

Figure S6: Chromatogram for samples stored at 25 °C under light exposure for 12 months. Peak at 1.739 min from cosolvent mixture, at 2.337 min from impurity A and the one at 3.443 min from PZQ

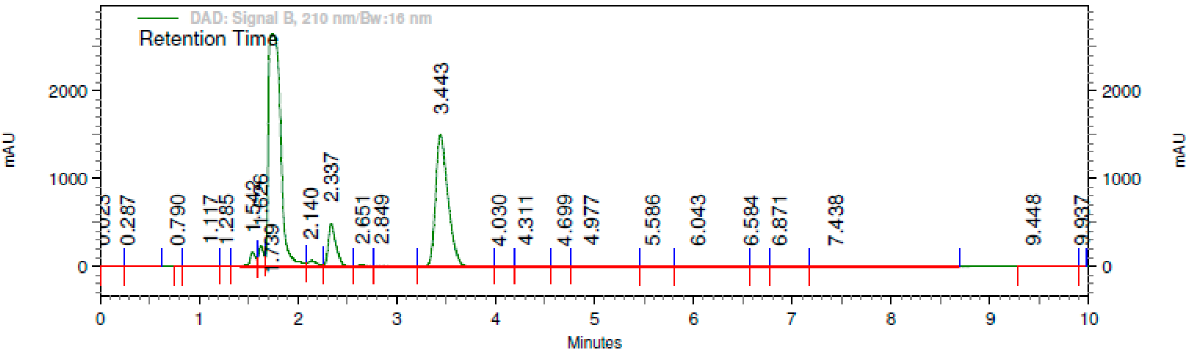

Table S7: Chromatographic information (retention time and peak area and height) for samples stored at 40 °C for 24 hours

DAD: Signal B, 210 nm/Bw:16 nm
Results

Retention Time	Area	Area %	Height	Height %
0.046	916	0.00	163	0.00
0.168	186	0.00	75	0.00
0.268	243	0.00	91	0.00
0.708	119	0.00	39	0.00
0.982	7838	0.01	1334	0.01
1.260	562258	0.73	85198	0.95
1.470	41379	0.05	8049	0.09
1.632	105259	0.14	25091	0.28
1.745	47562727	61.99	5568578	62.15
2.348	380482	0.50	40607	0.45
2.686	89783	0.12	10272	0.11
2.868	214989	0.28	17871	0.20
3.466	27658748	36.05	3197178	35.68
4.059	21862	0.03	1645	0.02
4.362	35955	0.05	1555	0.02
4.932	22699	0.03	896	0.01
5.542	4788	0.01	273	0.00
6.084	12036	0.02	616	0.01
6.637	189	0.00	51	0.00
8.520	3591	0.00	184	0.00
Totals	76726047	100.00	8959766	100.00

Figure S7: Chromatogram for samples stored at 40 °C for 24 hours. The peak at 1.745 min from cosolvent mixture and the one at 3.446 min from PZQ

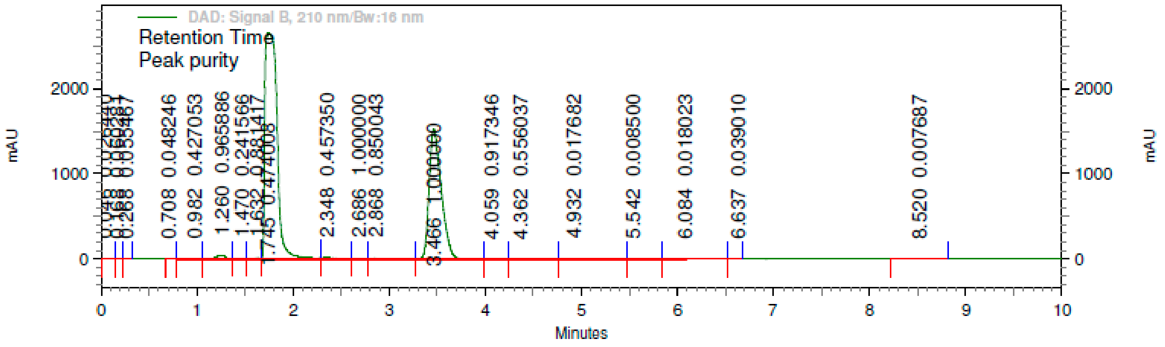

Table S8: Chromatographic information (retention time and peak area and height) for samples stored at 40 °C for 12 months

DAD: Signal B, 210 nm/Bw:16 nm Results Retention Time	Area	Area %	Height	Height %
0.046	769	0.00	145	0.00
0.174	200	0.00	64	0.00
0.297	215	0.00	75	0.00
0.966	15824	0.02	1710	0.02
1.244	594748	0.69	92974	0.87
1.373	17188	0.02	6533	0.06
1.543	1542074	1.80	361665	3.38
1.625	678550	0.79	201188	1.88
1.741	47534368	55.50	5560766	51.95
2.138	1117888	1.31	177321	1.66
2.334	5787111	6.76	1061252	9.91
2.653	166056	0.19	17773	0.17
2.843	200471	0.23	15184	0.14
3.439	27787645	32.44	3197366	29.87
4.033	27520	0.03	2181	0.02
4.322	36585	0.04	1934	0.02
4.918	84302	0.10	2365	0.02
5.705	1703	0.00	431	0.00
6.033	17544	0.02	1427	0.01
6.134	19188	0.02	1382	0.01
7.400	19242	0.02	915	0.01
Totals	85649191	100.00	10704651	100.00

Figure S8: Chromatogram for samples stored at 40 °C for 12 months. The peak at 1.741min from cosolvent mixture and the one at 3.439 min from PZQ

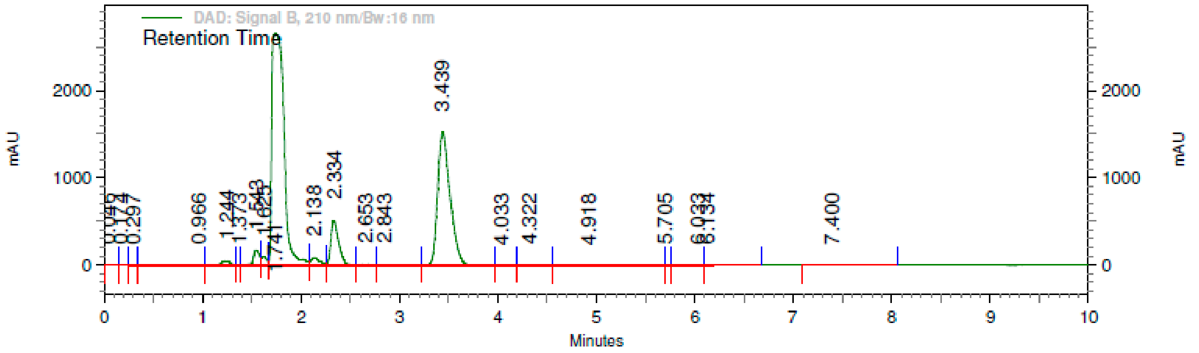

Supplement: Supplementary file 1 [file pharmaceutics-15-02050-s001.zip › pharmaceutics-2480844-supplementary.pdf]
